# Evaluation of an Online Learning Concept for Nursing Students in Caring for Patients with Dementia: Results of a Questionnaire Survey

**DOI:** 10.3390/nursrep13010009

**Published:** 2023-01-10

**Authors:** Adina Dreier-Wolfgramm, Anja Teubner, Katrin Kern

**Affiliations:** Department of Nursing and Management, Faculty of Business & Social Science, University of Applied Science (HAW) Hamburg, 20099 Hamburg, Germany

**Keywords:** nursing education, distance learning, nursing education research, dementia

## Abstract

Background: Nurses have key roles in caring for patients with dementia. To prepare them for demand-oriented nursing care, there is a need for education. An online course with a focus on healthcare needs assessment was designed. Methods: A quantitative study with a qualitative component using questionnaire-based interviews was implemented. The recruitment of students used the following inclusion criteria: (a) second-year students, and (b) absent from less than three lectures. Overall, *n* = 48 met the inclusion criteria. Twenty-eight students participated. Quantitative data were analyzed using descriptive statistics. Qualitative data were evaluated using Kuckartz’s content analysis with the software MAXQDA. Results: Nursing students judged the overall course structure as very good (M 1.36, SD 0.48). The learning aims were clearly defined (M 1.61, SD 0.68) and the learning content was adequately demonstrated (M 1.68, SD 0.67). The exercise on geriatric assessment promoted the internal learning process (M 1.67, SD 1.00). The online simulation training made the lectures’ contents easier to understand (M 1.86, SD 0.89). Correlations were determined, among others, between the course structure and the clear definitions of the learning aims (r^Sp^ 0.566, *p* = 0.002). Conclusions: The study describes the feasibility of the online course. To identify the impact on patients’ health and caregivers’ lives, further studies are needed.

## 1. Introduction

Dementia is a major public health concern in the older population. Currently, about 50 million patients are living with dementia worldwide. According to forecasts, the number will increase to more than 130 million patients by 2050 [1]. The majority live in the community [1]. As a result, various dementia care models have been developed for primary healthcare, which can be summarized as ‘collaborative care’ or ‘patient-centered-care’. The focus is on long-term systematic approaches to improving the management of the disease rather than treating the acute symptoms [2] and the aim is to address patients’ and caregivers’ complex healthcare needs and to enable multidisciplinary care [3,4,5]. Nurses have a key role in caring for patients with dementia and relieving caregivers. To prepare them for adequate demand-oriented nursing care provision and successful collaboration with other healthcare professionals, there is a need for education [6]. An analysis of present training shows that dementia is an important topic in nursing curricula. Nevertheless, it still focuses on treating specific symptoms (e.g., challenging behavior) [7], as well as diagnostic [8] and end-of-life care [9,10]. Programs are characterized by inconsistencies and gaps in professional dementia education [11]. Evidence shows that there are deficits in knowledge of dementia and inadequate skills in key elements of dementia care [12]. Dementia care nursing begins with a systematic assessment of the specific needs for healthcare services and issues involved in counseling.

Though there are a variety of nursing student training options related to dementia, none of them focus on a systematic assessment of patients’ healthcare needs. To overcome this gap, a learning concept for nursing students was developed. Due to the COVID-19 pandemic, face-to-face lectures and clinical training with patient simulations are a challenge [13,14,15]. Therefore, this concept was designed as an online course using the Microsoft Teams platform. It addresses exciting nursing care services for elderly patients and the systematic assessment of healthcare needs for patients with dementia using the so-called geriatric assessment. The focus is on nursing care for elderly patients in primary care. The online learning concept runs for one semester and is part of the module ‘Caring for elderly patients’ in the seven-semester nursing program. It comprises the following: (a) four theoretical lectures on caring for elderly patients with dementia; (b) the geriatric assessment, including an exercise on its use and application by nursing students; and (c) online simulation training.

The four theoretical lectures deal in detail with the specifics of caring for patients with dementia, as well as the settings and nursing-related healthcare services that exist for these patient groups. This is followed by the introduction of the geriatric assessment, which is a multidimensional comprehensive evaluation of the health status of elderly patients [16] that covers eight areas: (1) activities of daily living, (2) cognition, (3) psychological state, (4) social situation, (5) sensory functions, (6) mobility, (7) nutritional status, and (8) incontinence [16]. The nursing students were divided into groups of four or five students to become more familiar with the geriatric assessment concept including the relevant assessment instruments. The student groups comprehensively used the geriatric assessment instruments by themselves. The online simulation training includes a briefing, the geriatric assessment in an online simulation, and a debriefing, and lasts 90 min: 15 min each for the briefing and debriefing, and 60 min for the geriatric assessment. Not all instruments (e.g., hand-force test or hearing test) are suitable for an online interview. Therefore, the nursing students selected the following instruments to systematically identify the healthcare needs: (1) activities of daily living (Barthel index, IADL); (2) cognition (MMSE, clock test); (3) psychological state (GDS); (4) social situation (family status and living situation); (5) sensory functions (Visus test, hearing test, pain scale); (6) mobility (Performance-Oriented Mobility Assessment by Tinetti, Timed Up-and-Go Test, hand-force test); (7) nutritional status (MNA); and (8) incontinence (situational awareness questionnaire). The patient with dementia was a standardized simulation performed by a professional actor who had also completed a nursing education program. The results of the online assessment provided the basis for the nursing students to develop a healthcare plan for patients with dementia.

Currently, there is a lack of knowledge about online nursing education programs focusing on the assessment of the healthcare needs of patients with dementia. To better prepare nursing students for the systematic assessment of the healthcare needs of patients with dementia and specify the impacts of the online learning concept, the Elaine Study (evaluation of a nursing simulation learning concept caring for patients with dementia) was conducted. It addressed the following research question: How do nursing students feel about the online learning concept regarding (a) the theoretical lectures; (b) the exercise on the geriatric assessment; and (c) the online simulation training to assess patients’ healthcare needs? The aim was to examine the feasibility of the online education concept, as well as identify its strengths and weaknesses to provide an optimized online nursing education program in the future.

## 2. Materials and Methods

The present analyses are based on data derived from the Elaine Study, which is a quantitative study design with a qualitative component using questionnaire-based interviews.

### 2.1. Participants

The recruitment of nursing students for the study was guided by the following inclusion criteria: (a) second-year nursing students participating in the module ‘caring for elderly patients’, (b) less than three lectures had been missed. Both criteria were chosen to ensure that the nursing students enrolled in the study were partaking in the course in its recently developed online format for the first time. Therefore, it was important that the students had received an overall impression of the whole online education program. This required almost perfect attendance in the course. Overall, 48 met the inclusion criteria. At the beginning of the 2021 summer semester, students were informed about the study in the first lecture by the principal investigator. Students were given the opportunity to ask questions regarding the design and the order of the study. All 48 nursing students expressed an interest in participation. The project information and written informed consent were mailed by the PI to the 48 nursing students. Then, they had 14 days to decide on their participation in the study. If the completed and signed written informed consent forms were sent by mail to the PI, the students were enrolled in the Elaine Study. Twenty students rejected participation. Reasons for non-participation included (a) lack of time (*n* = 15); (b) preparing for upcoming examinations (*n* = 4); and (c) being abroad during the data collection phase (*n* = 1). Over the course of the study, none of the students dropped out.

### 2.2. Data Measurement

This study is one of the first explorations of the evaluation of an online nursing education program focusing on the systematic assessment of the healthcare needs of patients with dementia. No valid and reliable instrument in the form of a standardized questionnaire was available. Therefore, a questionnaire was developed instead. It was based on existing evaluation instruments for academic education programs and online lectures from the Philipps University of Marburg.

The quantitative mailed questionnaire consisted of three dimensions: (a) a theoretical introduction to nursing healthcare services and the geriatric assessment (lecture evaluation: 10 items, docent evaluation: 11 items); (b) the geriatric assessment exercise (10 items); and (c) online simulation training (in MS Teams: 21 items, docent evaluation: 3 items, patient simulation evaluation: 9 items). A five-point Likert scale with the response categories ‘1 = strongly agree’, ‘2 = partly agree’, ‘3 = neither agree nor disagree’, ‘4 = partly disagree’, and ‘5 = disagree’ was used.

The instrument ended with semi-structured questions with respect to the identified positive aspects and suggestions for improvement, as well as socio-demographic data (age, gender, number of students missing lectures, professional experience in caring for patients with dementia).

The survey instrument was pretested in a small group with representatives of the target group (*n* = 6 nursing students, *n* = 3 females) who did not participate in the subsequent data collection. The aim was to identify the acceptance and practicability of the questionnaire and the clarity of the questions and response categories, as well as determine suggestions regarding a lack of relevant issues being addressed. The pretest was carried out via a questionnaire mailed to the participants. They completed the questionnaire by themselves and made written notes on the document. The completed instruments were sent to the study center. Two study members analyzed the written notes and made adaptions concerning linguistic changes and individual terms. Comprehensive adjustments regarding size reduction, response format, or structural changes were not required. Finally, the questionnaire, which consisted of 3 dimensions with 74 items, was used for the subsequent quantitative survey. Missing values occurred less frequently (<5%). This demonstrates the satisfactory and acceptable nature of the questionnaire.

### 2.3. Data Collection

The data collection was carried out between May and June 2021. In total, 28 questionnaires were sent to nursing students after completing the course at the end of the 2021 summer semester. All students received the questionnaire by mail at the end of May and completed it by the end of June 2021. Completion took 15–20 min. Completed questionnaires were sent back to the study center by 28 nursing students.

### 2.4. Data Analysis

For analysis, the data were verified and documented in a PDF database and then transferred to the software SPSS (IBM Statistics 26, 2020). The quantitative data of the mailed questionnaire were analyzed using descriptive statistics. To identify the relationships between the evaluations of the lectures and the professional experiences of the students in caring for patients with dementia, the Chi-squared test was used. For analyzing the possible relationships between the different aspects of the evaluations of the lectures, the Spearman correlation was used.

Collected qualitative questionnaire data from semi-structured questions regarding positive aspects and suggestions for improvement were transcribed and analyzed using Kuckartz’s proposed qualitative content analysis [17] with the MAXQDA Version 20.4.1 software (VERBI GmbH, Berlin). Following analysis, several steps were performed: (1) initiating textual work (highlighting important text passages, writing memos), (2) development of major thematic categories, (3) coding questionnaires by major thematic categories, (4) sorting text passages into the same major thematic categories, (5) inductive development of subcategories based on the questionnaires, (6) coding of the complete textual material by the differentiated category system, and (7) category-based analysis and presentation of the results [17].

Two study team members coded semi-structured interview questions according to the consensual coding approach. First, both team members coded separately. Then, the two deductively developed resulting category systems were compared for similarities and differences. In the case of differences, the codes were discussed within the whole study team and modified if all team members agreed. This approach caused an extension of the category system. In conclusion, a system with categories, sub-categories, and codes based on the code systems of both coders was developed.

## 3. Results

### 3.1. Participants’ Characteristics

Most of the 28 nursing students (response rate: 58.3%) were female (*n* = 25; 89.3%) and more than half (53.6%) had professional experience in caring for patients with dementia just before the course began. The average age was 21.85 years (SD 2.83). Almost 90 percent (*n* = 25) of the students participated in all lectures of the module.

### 3.2. Course Structure

Generally, it was observed that the students judged the overall course structure as being very good (M 1.36, SD 0.48) (see Table 1 below). The learning aims were clearly defined (M 1.61, SD 0.68), the learning content was adequately demonstrated (M 1.68, SD 0.67) and the teaching methods contributed to their understanding (M 1.63, SD 0.62). The majority of nursing students ‘partly agree’ (*n* = 17; M 2.21, SD 0.99) that the increase in individual learning ability was high and there was also an increased interest in caring for patients with dementia (M 2.32, SD 1.09).

No significant evidence was found of a link between students’ course structure evaluations and their former professional experience in caring for patients with dementia by the Chi-squared tests. In contrast, a specific comparison of the course evaluation aspects identified significant associations (see Table 2 below). Four positive significant correlations were determined between the course structure and (1) clear definitions of the learning aims (r^Sp^ 0.566, *p* = 0.002), (2) presentations and references being made available (r^Sp^ 0.514, *p* = 0.005), (3) teaching methods contributing to understanding (r^Sp^ 0.535, *p* = 0.004), and (4) an increase in individual learning ability (r^Sp^ 0.573, *p* = 0.001).

In addition, there were numerous positive significant correlations between clearly defined learning aims and presentations and references being made available (r^Sp^ 0.619, *p* = 0.000), as well as between learning content that was dealt with at an appropriate pace and the (1) increase in individual learning ability (r^Sp^ 0.632, *p* = 0.000), (2) the up-to-date teaching content (rSp 0.588, *p* = 0.002), and (3) the increased interest in caring for patients with dementia (r^Sp^ 0.594, *p* = 0.001).

### 3.3. Exercise on Geriatric Assessment

Table 3 below highlights the nursing students’ evaluation results for the geriatric assessment exercise, which shows that they predominantly judged it as being positive. It had a clearly defined structure (M 1.50, SD 0.51), built on previous knowledge (M 1.68, SD 0.77), and, therefore, promoted the internal learning process (M 1.67, SD 1.00). Along with the belief that the geriatric assessment allowed sufficient time (M 1.21, SD 0.41), nursing students concluded that their interest in the lecture topics increased and there was an improvement in their ability to carry out the learning content in nursing care practice.

### 3.4. Online Simulation Training

For the final online simulation training to assess the healthcare needs of patients with dementia, nursing students illustrated that there were both positive and negative aspects to this learning format (see Table 4 below). Students stated as being positive the clearly defined structure (M 1.39, SD 0.56); sufficient time for the online simulation training (M 1.08, SD 0.27); and that it built on previous knowledge (M 1.92, SD 0.93). The support of the online simulation format in making lecture content easier to understand was mostly evaluated as ‘partly agree’ (M 1.86, SD 0.89). On the contrary, the aims of providing specific practical skills, enhancing the understanding of complex issues, and increasing nursing students’ learning motivation were partially achieved. Nursing students predominantly evaluated these three aspects as ‘neither agree nor disagress’.

Positively significant evidence was found for correlations between the fact that the online simulation training increased nursing students’ learning motivation and the fact that it built on previous knowledge (r^Sp^ 0.533, *p* = 0.004), as well as made lecture content easier to understand (r^Sp^ 0.536, *p* = 0.004).

### 3.5. Positive Aspects and Implications for Improvement

Nursing students mentioned more positive aspects than suggestions for improvement (see Table 5). The following positive responses were recorded with respect to (1) the structure of the lectures; (2) the self-determined learning; (3) the docent; and (4) the online simulation training. The structure of the course and the order of (a) theoretical lectures, (b) a geriatric assessment exercise, and (c) online simulation training were described as very positive. 

Schedule planning without feeling that one was learning under time pressures was rated very positively. Therefore, a pleasant learning atmosphere was successfully achieved. Individual lectures were well prepared. The variance in the course content better balanced the previously used lecture structure, consisting of classroom teaching and self-study. The possibility to present previous practical experience and compare these cases among the group’s members were also stated as being positive aspects.

A nursing student concluded that *“The lectures were well-organized. We always knew what the next steps are, and we were able to manage weekly work assignments”.* (FB 0002:7–8)

The opportunity to use the geriatric assessment was a further positive aspect according to students. It enabled learning at one’s own pace, allowed for further individual engagement with the learning content, and promoted a growing interest in caring for patients with dementia. The docent’s kindness toward nursing students, the given opportunity to voice requests for further information, and the mutual sharing of previous professional experience in caring for patients with dementia were positively emphasized by the nursing students. For the online simulation training, the nursing students felt well prepared for the theoretical lectures. In addition, they were allowed sufficient time to prepare.

The nursing students appreciated the opportunity to test their own skills *“without any pressure. I was shown my own practical competences clearly, and current existing deficits as well.”* (FB 0030: 64–65)

The use of a simulation patient was very welcome and enhanced practical applications.

In conclusion, a nursing student summarized feeling *“well-prepared for the first practical experience in caring for elderly patients, while the knowledge taken from the lecture will provide the requisite safety in handling this patient group”.* (FB 0043:73–75)

There were major challenges in terms of technology (e.g., poor internet connection). Online discussions were difficult to implement, and it was suggested that some students inadequately participated in lectures.

One nursing student concluded that *“a remarkable difference is noticeable between classroom learning and online education”.* (FB 0015: 116.117)

This resulted, among other things, in the inability to apply the geriatric assessment instruments. Therefore, a reduction in the number of team members from four or five to two was instead proposed.

## 4. Discussion

The Elaine Study aimed to examine the feasibility of the online course concept and identify its strengths and weaknesses. Overall, the nursing students confirmed the format’s feasibility and stated that there was an increase in their individual learning abilities and interest in caring for patients with dementia. Bickford et al. (2019) reported similar results and concluded that nursing students had a broad understanding of dementia [7]. They gained substantial knowledge and understood the importance of early disease detection and needs-based treatment [18]. Other study results showed an increase in students’ positive attitudes toward caring for patients with dementia. A student’s age, academic year, previous training, and practical experience in caring for patients with dementia were shown to have a beneficial effect on their attitude [19]. Subsequently, students developed increased confidence in caring for people with dementia after completing a specific related education program [19,20,21]. Strengthening nursing students’ self-efficacy was described by Takeuchi et al. [22]. In the long run, a positive contribution to the improvement of dementia care can be expected [7]. A recent meta-analysis confirmed these findings and underlined the effectiveness of simulation training in preparing nursing students for caring for patients with mental disorders [23]. Nevertheless, there is a need for further studies on learning outcomes to be able to develop and implement strategies for simulation-based undergraduate nursing education in the future [24].

The COVID-19 pandemic posed a huge challenge to the education system. Most lectures were delivered fully online. Buléon et al. (2022) investigated the proportion of online simulation training during the COVID-19 pandemic in 32 countries (with a total of 618 participants) and found that the majority (70%) conducted simulations at a distance [25]. The online education course in the Elaine Study showed that nursing students were satisfied with this digital program. Li et al. (2021) reinforced these findings and identified the key factors that affected students’ attitudes to online education. These included (a) a teacher’s professional title; (b) a student’s year of study; and (c) the current place of residence [14]. Additional factors have been identified and comprise (1) the choice of online teaching mode; (2) the platform or technology; (3) faculty preparedness; and (4) the learner’s motivation and expectations [15]. The supplementary evaluation results of the Elaine Study demonstrated that there was no connection between students’ course evaluations and their previous professional experience in caring for patients with dementia. For this purpose, Swaminathan et al. (2021) addressed the willingness of students to learn through online formats and concluded that previous experience with digital courses and the perceived advantages thereof might influence nursing students’ readiness to participate [26].

For the online simulation training, the nursing students emphasized that they preferred to have sufficient time to prepare. As a consequence, students’ previous knowledge was built upon. Schedule planning without feeling that one was learning under time pressures was rated very positively. Using the online format for the simulation training supported the core aim to make the content of lectures easier to understand. Further advantages of this approach were described by Taylor et al. (2021). They reported on an online simulation training experience that presented no risk to patient safety [27]. The nursing students taking part in the Elaine Study confirmed this finding and mentioned the possibility to learn without any pressure by having the opportunity to exercise their own practical competencies, as well as identifying their current existing deficits in a secure learning environment, as being significantly positive. As a complement to this, Meyer et al. (2020) obtained comparable results based on their own study including the finding that online simulation training enhances traditional teaching methods and helps students to understand the experiences of people living with dementia [28]. Furthermore, students were inspired to reflect on their actions during the simulation training [28,29,30]. The nursing students taking part in the Elaine Study, therefore, noted feeling better prepared for future practical experience. As a consequence, Meyer et al. proposed a modified transformative learning process regarding simulation training for healthcare students to create the best possible prerequisites for optimum dementia care in the future [28]. In the opinion of Taylor et al., these mentioned advantages are important fundamentals for enhancing the learning experience and creating effective, efficient clinicians in the future [27].

The Elaine Study has its limitations. First, the use of purposive sampling for the nursing students may be highly prone to selection bias. The definition of clear inclusion criteria should offset this potential bias. Second, no standardized questionnaire was available. Therefore, a questionnaire was developed. It was based on existing evaluation instruments for academic education programs and online lecture evaluation instruments from the Philipps University of Marburg. In addition, a pretest was conducted, which resulted in linguistic adaptions, but no comprehensive adjustments were needed. Third, there might be a risk of bias related to the possibility that the responses of the nursing students were given according to social desirability. This occurs when sensitive issues are addressed in questionnaire-based interviews [31]. However, the Elaine Study evaluated the online learning concept regarding its structure and learning content and no sensitive questions were involved. The used questionnaire was based on existing and implemented evaluation instruments for academic education programs and online lectures from the Philipps University of Marburg. A five-point Likert scale with the response categories ‘1 = strongly agree’, ‘2 = partly agree’, ‘3 = neither agree nor disagree’, ‘4 = partly disagree’, and ‘5 = disagree’ was used. The results of the pretest suggest that there was no bias. Nevertheless, generalization to the whole nursing student population would be limited. 

The increase in the interest and confidence in caring for patients with dementia, as well as the impact on patients in daily nursing care practice, need to be analyzed in detail in the future. Due to the COVID-19 pandemic, online simulation training has become an important teaching tool. Nevertheless, the benefits for nursing students in comparison to in-person simulation training, including the impact on nursing practice, need to be evaluated more comprehensively in future studies.

## 5. Conclusions

In summary, this study describes the feasibility of an online learning concept for nursing students in caring for patients with dementia, with a focus on the assessment of the latter’s healthcare needs. The nursing students judged the overall course structure as being very good and appreciated the opportunity for online simulation training as a medium with great potential as an alternative learning method during the pandemic. Therefore, nursing students’ interest in caring for patients with dementia increased, as did their individual learning abilities. There remain various barriers to the sustainable implementation of this online learning format, including, in particular, technical issues, the inadequate participation of some students, and the limited opportunities to employ specific learning methods (e.g., critical discussions). As a consequence, further studies with a greater number of nursing students are needed to identify the ideal learning outcomes in more detail. The actual impact on patients’ health and caregivers’ lives, as well as students’ professional competence, need to be studied in controlled prospective studies in the future.

## Figures and Tables

**Table 1:** Evaluation of the course structure.

Course Structure Aspects	Mean (SD)
Course structure was very good.	1.36 (0.48)
Learning aims were clearly defined.	1.61 (0.68)
Learning content was properly demonstrated.	1.68 (0.67)
Presentations and references were made available.	1.75 (0.88)
Learning content was dealt with at an appropriate pace.	1.74 (0.94)
Learning content was too extensive.	3.86 (0.97)
Teaching methods contributed to my understanding.	1.63 (0.62)
Teaching content was up to date.	1.48 (0.64)
Increase in individual learning ability was high.	2.21 (0.99)
The lectures increased my interest in caring for patients with dementia.	2.32 (1.09)

1 = strongly agree, 2 = partly agree, 3 = neither agree nor disagree, 4 = partly disagree, 5 = disagree.

**Table 2:** Spearman correlation ‘course structure aspects’.

Course Structure Aspects	r^SP^	*p*-Value
Course structure was very good. * Learning aims were clearly defined.	0.566	0.002
Course structure was very good. * Presentations and references were made available.	0.514	0.005
Course structure was very good. * Teaching methods contributed to my understanding.	0.535	0.004
Course structure was very good. * Increase in individual learning ability was high.	0.573	0.001
Learning aims were clearly defined. * Presentations and references were made available.	0.619	0.000
Learning content was dealt with at an appropriate pace. * Increase in individual learning ability was high.	0.632	0.000
Learning content was dealt with at an appropriate pace. * Teaching content was up to date.	0.588	0.002
Learning content was dealt with at an appropriate pace. * The lectures increased my interest in caring for patients with dementia.	0.594	0.001

**Table 3:** Evaluation results of the independent exercise on geriatric assessment.

Exercise on Geriatric Assessment	Mean (SD)
had a clearly defined structure.	1.50 (0.51)
promoted the internal learning process.	1.67 (1.00)
was easy for me.	2.14 (0.89)
took sufficient time.	1.21 (0.41)
built on previous knowledge.	1.68 (0.77)
had a lot of disruptions so I could not learn efficiently.	4.23 (1.07)
hardly increased my knowledge.	3.74 (1.09)
hardly changed my interest in the lecture’s theme.	3.59 (1.15)
…hardly changed my abilities to carry out learning in practice.	3.70 (0.99)

1 = strongly agree, 2 = partly agree, 3 = neither agree nor disagree, 4 = partly disagree, 5 = disagree.

**Table 4:** Evaluation of the online simulation training.

The Online Simulation Training	Mean (SD)
had a clearly defined structure.	1.39 (0.56)
took sufficient time.	1.08 (0.27)
had a low level of difficulty.	3.00 (0.93)
built on previous knowledge.	1.92 (0.93)
made a significant contribution to understanding the content of the lecture.	2.00 (0.96)
provided specific practical skills.	2.64 (0.99)
enhanced my understanding of complex issues.	2.59 (0.93)
enhanced my ability to carry out the content in practice.	2.00 (0.90)
contributed to ensuring that the course was varied and interesting.	1.00 (0.79)
increased my interest in caring for patients with dementia.	2.00 (0.95)
increased my learning motivation.	2.46 (1.20)
hardly changed my knowledge.	4.00 (0.88)
hardly increased my capacity for application to professional practice.	4.00 (0.99)
I prefer to learn face to face.	2.46 (1.39)
**Virtual learning environment**	
acoustics were very good	2.00 (0.94)
technical quality of image transition was very good.	2.00 (0.92)
the learning environment was realistic.	4.00 (1.28)
online briefing and debriefing were very good.	2.00 (0.81)
**The simulation patient**	
was kind and respectful when dealing with students.	1.00 (0.26)
acted in a competent and professional manner.	1.00 (0.46)
embodied the role authentically.	1.00 (0.60)
gave a realistic demonstration.	1.00 (0.69)
adequately tested students’ professional capabilities.	2.00 (1.12)
gave students a sense of working with a real-life patient.	2.00 (1.16)
gave helpful feedback on students’ performance.	1.00 (0.70)
sufficiently addressed further questions during debriefing.	1.00 (0.54)

1 = strongly agree, 2 = partly agree, 3 = neither agree nor disagree, 4 = partly disagree, 5 = disagree.

**Table 5:** Qualitative evaluation results—category system.

**Positive aspects**
lecture organization
structure and running of single lectures
preparation of lectures
security of the subsequent learning content
lecture schedule planning
good learning atmosphere
interesting and relevant learning content
ideal variety of theoretical and practical learning
theoretical lectures—exercise—simulation training
exchange of practical experiences
self-paced learning
independent practice of the geriatric assessment
learning to one’s own rhythm
learning at own pace
individual examination of learning content
indication of interest in caring for patients with dementia
docent
kindness
interacted openly with students and learning content
possibility for further inquiries and additional questions
commented on previous professional experience
online simulation training
good preparation from theoretical lectures
possibility to enhance own skills
being allowed to make mistakes
high learning outcomes
standardized patient simulation
realistic exercise
strong practical relevance
identification of students’ existing expertise and deficits
**Challenges**
technical problems
lack of internet access
online learning
implementing online group discussions
experience exchange online
temporarily limited learning motivation
low participation by a small number of students in the groups
geriatric assessment exercise
use of instruments
**Implications for improvement**
establishment of two nursing student teams

## Data Availability

The datasets used and analyzed during the current study are available from the corresponding author Adina Dreier-Wolfgramm (adina.dreier-wolfgramm@haw-hamburg.de).
